# Life long follow up and management strategies of patients living with native livers after Kasai portoenterostomy

**DOI:** 10.1038/s41598-021-90860-w

**Published:** 2021-05-27

**Authors:** Patrick Ho Yu Chung, Edwin Kin Wai Chan, Fanny Yeung, Albert Chi Yan Chan, Jennifer Wai Cheung Mou, Kim Hung Lee, Judy Wing Suet Hung, Michael Wai Yip Leung, Paul Kwong Hang Tam, Kenneth Kak Yuen Wong

**Affiliations:** 1 Division of Paediatric Surgery, Department of Surgery, Queen Mary Hospital, Li Ka Shing Faculty of Medicine, The University of Hong Kong, Pok Fu Lam, Hong Kong, SAR; 2 Division of Pediatric Surgery and Pediatric Urology, Department of Surgery, Prince of Wales Hospital, The Chinese University of Hong Kong, Shatin, Hong Kong, SAR; 3 Division of Liver Transplantation, Department of Surgery, Queen Mary Hospital, Li Ka Shing Faculty of Medicine, The University of Hong Kong, Pok Fu Lam, Hong Kong, SAR; 4 Department of Surgery, Hong Kong Children’s Hospital, Ngau Tau Kok, Hong Kong, SAR

**Keywords:** Gastroenterology, Health care

## Abstract

We present a 37 years’ experience in the management of biliary atresia (BA) and discuss long-term complications after Kasai portoenterostomy (KPE). A retrospective territory-wide study from 1980 to 2017 on 231 patients with open KPE from three tertiary paediatric surgical centres was performed. Outcome parameters were clearance of jaundice (COJ), native liver survival (NLS) and long-term complications. Factors affecting the operative outcomes were analyzed. The median duration of follow up was 17.5 (IQR: 13.5–22) years. Over 66% of patients became jaundice-freed at 1 year after KPE. Seventy patients (30.3%) received liver transplant (LT) at a median age of 6.2 (IQR: 4.3–8.4) years. The NLS rates at 10 and 20 years were 70.7% and 61.5% respectively with no significant change over the study period. The median age at KPE was 59 (IQR: 49–67) days. KPE performed before 70 days was associated with higher odd ratios for successful drainage but the age of KPE did not have an impact on the long-term NLS. Among all native liver survivors (n = 153), the median bilirubin level was 24 (IQR: 16–36) µmol/L. Portal hypertension (PHT) and recurrent cholangitis were found in 51.6% and 27.5% of them respectively. With a vigilant follow up program, more than 60% of BA patients could remain stable with the disease and achieve long-term survival without LT. Although cholestasis, portal hypertension and recurrent cholangitis are common in long-term NLS, with a comprehensive follow management strategy, they do not always necessitate LT. Our study serves as an example for countries where deceased donor organs are scarce due to very low donation rate.

## Introduction

Biliary atresia (BA) is a progressive fibrosclerosing disease of the biliary tract and affects all ethnicities with a noticeably higher incidence in the Asia–Pacific region^1^. Kasai portoenterostomy (KPE) is by far the most widely accepted primary treatment with a variable outcome. Liver transplant (LT) is regarded as the salvage treatment when KPE fails to restore biliary drainage. KPE is also labelled as failure when patients develop complications related to recurrent cholangitis, portal hypertension (PHT) and hepatic dysfunction which could happen in 60% of BA patients^2^. These complications are often indications for LT. Literatures from various studies have reported that early drainage rate is in the range of 50% to 60% and only less than half of the patients could remain transplant-free after KPE^1,3–5^. In addition, it was estimated that around half of the LT will be performed before the age of 2^6^. Although LT is a potential treatment for these complications, transplant recipients need to face the problems associated with an ultra-major operation and the life-long use of immunosuppressants. This could impair the immune system, leading to recurrent infection and most severely, haematological malignancy^7^. Even worse, this notorious side-effect is more pronounced in children. In some countries, another major hurdle to LT is the low organ donation rate. Thus, eliminating or deferring the need of LT for as long as possible is a legitimate goal for BA treatment. The main purposes of this study were (1) to describe our territory-wide experience in managing BA and report the treatment outcomes of KPE based on 37 years’ follow up data; and (2) to present the long-term problems encountered by native liver survivors and discuss management strategies other than LT.

## Methods

### Study design and patients

This was a retrospective regional-based study conducted in the only three tertiary paediatric surgical centres performing KPE in Hong Kong. A list of BA patients receiving treatment between 1980 and 2017 was retrieved and their medical records were reviewed. This study has been approved by the University of Hong Kong/Hospital Authority Hong Kong West Cluster Institutional Review Board (HKU/HA HKW IRB number: UW20-156) and was performed in accordance with the ethical standards in the Declaration of Helsinki. Informed consent from parents and/or legal guardians for study participation have been obtained.

### The management strategies of BA in Hong Kong

In Hong Kong, the three centres broadly adopted a common approach for BA over the years. The diagnosis of BA was established by surgical exploration + /- cholangiogram and liver biopsy. KPE remained the preferred primary treatment option for BA, except when the patients presented over 100 days and/or surgical exploration revealed a grossly cirrhotic liver. In those situations, the chief surgeons decided to proceed with KPE or not and for the latter case, the patients would be referred for LT. All KPEs were performed by an experienced paediatric surgeon who had completed fellowship training for more than 5 years and assisted KPE for at least 10 times. In this way, surgical expertise is guaranteed. To ensure an adequate training of the next generation, younger surgeons were also present during the operations.

KPE was performed by conventional open approach according to the original principle with minor technical variations, except 16 laparoscopic KPEs were performed by a single surgeon in one of the centres from 2002 to 2006. The operation started with a right subcoastal or upper transverse abdominal incision. The liver was either completely mobilized and everted out of the wound or remained attaching with the ligaments. The right and left portal veins were used as the reference landmarks for hilar dissection, which was followed by a 30 to 45 cm Roux-en-Y biliary reconstruction. Porto-enterostomy was anastomosed using 5/0 or 6/0 absorbable sutures.

Post-operatively, enteral feeding was resumed once the bowel function had returned. Ursodeoxycholic acid, fat-soluble vitamins and antibiotics were prescribed. Since 2004, 169 patients have received oral steroid as the adjuvant therapy after KPE with variations in the duration and dosage among the three centres. After discharge, life-long follow up was conducted by the primary surgical team for every 3 to 6 months. During each visit, complete blood count and liver function were checked. A more frequent visit would be scheduled if the liver function was abnormal or in the presence of complications necessitating a close monitoring. The clinical manifestations of PHT were actively screened. Splenomegaly was detected by clinical examination and ultrasonography. Oesophago-gastric varices (OGV) were managed by endoscopic sclerotherapy or banding. Medications for PHT were prescribed to patients with symptomatic PHT after discussion of their potency and potential side-effects. All patients admitted with fever and raised bilirubin level were promptly treated as cholangitis with at least two weeks of antibiotics after full septic work up. Radiological investigations were performed to look for liver abscesses/cysts or other structural anomalies in resistant cases. A central venous catheter would be inserted if more than 4 weeks of antibiotics was expected. Patients with BA-related complications admitted to district hospitals other than these three paediatric surgical centres would be transferred back to the parent team for management. The paediatric LT programme started in 1993 and both living as well as deceased donor LT were performed. The decision of LT was jointly reviewed by the paediatric surgical and the LT teams.

### Registered data and outcome measurements

The medical records were retrieved using ICD-9 coding ‘751.61: Biliary atresia’; ’51.37: Kasai portoenterostomy’ and ’50.59: Transplant of liver’. Demographic information, peri-operative details, clinical and laboratory data including serum bilirubin and albumin level; international normalized ratio (INR) and platelet count were extracted. In all patients, the outcome measurements were COJ (total serum bilirubin level < / = 20 µmol/L) and native liver survival (NLS) rates after KPE. NLS was defined as survival with own liver and has not been listed for LT at the time of writing. For native liver survivors, additional analysis included the assessment of growth and liver function. The following complications were evaluated: (i) hypersplenism which was defined by a clinically palpable spleen/spleen length above age-specific value in abdominal ultrasound and platelet count < 150 × 10^9^/L; (ii) OGV of any grade detected during surveillance or emergency endoscopy; and (iii) recurrent (> 1 episode) cholangitis. An episode of cholangitis was defined by the presence of fever (core body temperature > 38.5 degree Celsius) and bilirubin level > 20 µmol/L on two consecutive blood samples requiring intravenous antibiotics treatment. An episode that required more than 2 weeks of antibiotics or intervention was regarded as severe cholangitis. In this study, the exclusion criteria for outcome analysis were (i) incomplete medical record or laboratory data; (ii) loss of follow up data for more than 3 consecutive years; (iii) KPE performed by laparoscopic approach and iv) LT as the primary procedure.

### Statistical analysis

Scientific analysis was performed with a standard statistical package (Windows, version 26.0; SPSS Inc., Armonk, NY, USA). Categoric variables were compared with Chi-square test. Continuous variables were reported as medians (interquartile range) and compared with Kruskal–Wallis test. NLS was estimated with Kaplan–Meier analysis. Logistic regression analysis was performed to identify factors associated with COJ at 1 year after KPE. A p-value of less than 0.05 was considered to be statistically significant.

### Human transplantation research declaration

NO organs/tissues were procured from prisoners. Organ procurement for liver transplantation in this study was performed by the Division of Liver Transplantation, Department of Surgery, Queen Mary Hospital, The University of Hong Kong.

## Results

### Study population

**Figure 1:**
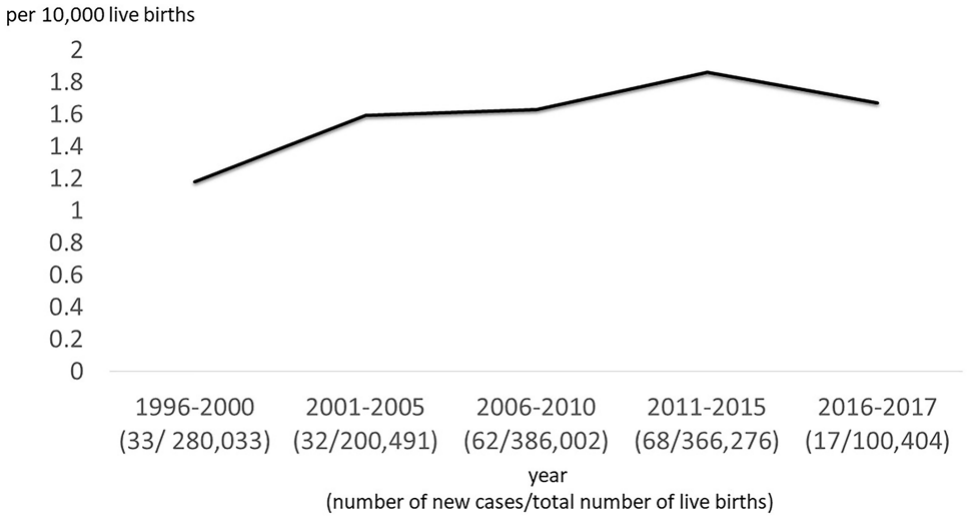
The incidence of BA (per 10,000 live birth) in Hong Kong from 1996 to 2017 .

**Figure 2:**
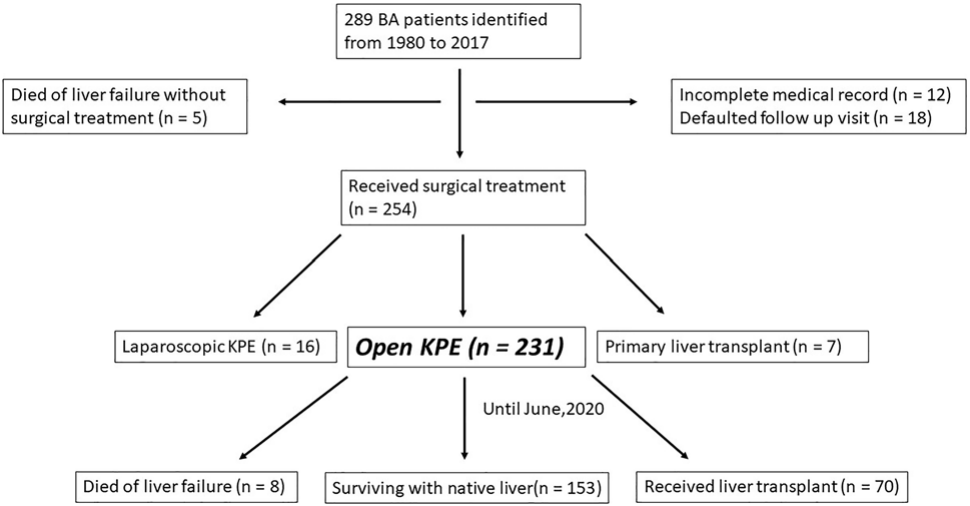
Flowchart of patient recruitment in this study. Ultimately, 231 BA patients with open KPE were analyzed for their clinical outcomes.

During the study period, there were 289 BA patients identified with more female than male (F:M = 168:121). Between 1996 to 2017, the annual incidence of BA in Hong Kong ranged from 1.18 to 1.86 per 10,000 live births (Fig. 1). Fifty-eight patients were excluded from the this study because of (i) incomplete medical record (n = 12); (ii) follow up visit defaulted (n = 18); (iii) laparoscopic KPE (n = 16) and (iv) LT as the primary procedure (n = 7). Furthermore, 5 patients who died of liver failure without any surgical treatment were also excluded. As a result, 231 patients with conventional open KPE as the primary surgical procedure were included (Fig. 2). The majority of them (98%) suffered from type III BA and 17 patients had syndromic association including Biliary Atresia Splenic Malformation (BASM). The median age at KPE was 59 (IQR: 49–67) days. In this study, all patients were followed up at their respective centres and the median duration of follow up was 17.5 (IQR: 13.5–22.0) years. The demographic data were summarized in Table 1.

**Table 1:** Demographic data and clinical outcomes of 231 BA patients with open KPE performed in Hong Kong between 1980 and 2017.

Patient characteristics	Number (%) or median (IQR)
**Sex**	
Male	97 (42.0%)
Female	134 (58.0%)
**Associated major anomalies**	
Yes	17 (7.4%)
No	214 (92.6%)
Age at KPE (days)	59 (49–67)
**Age at KPE**	
< 51 days	60 (25.9%)
51 to 60 days	63 (27.3%)
61 to 70 days	57 (24.7%)
> 70 days	51 (22.1%)
**Use of adjuvant steroid since 2004**	
Yes	169 (73.2%)
No	62 (26.8%)
Duration of follow up (years)	17.5 (13.5–22.0)
Jaundice clearance*	
1 year after KPE	154 (66.7%)
2 years after KPE	149 (64.5%)

*Failure of clearance of jaundice (COJ) is defined as serum bilirubin > 20 µmol/L or transplanted.

### Post-KPE outcomes (n = 231)

#### Clearance of jaundice

COJ was defined as serum total bilirubin level < / = 20 µmol/L. Patient who have received LT were considered as failure of COJ as none of them had a normal bilirubin level prior to LT. The COJ rates at 1 and 2 years after KPE were 66.7% and 64.5% respectively. By logistic regression analysis, performing KPE before 70 days was associated with higher odds ratios for normal bilirubin level at 1 year after KPE (Table 2).

**Table 2:** Percentage and odds ratio from logistic regression for the relation between different clinical variables and clearance of jaundice (COJ) at 1 year after KPE (n = 154/231, 66.7%).

	Number of patients	Number and % of patients achieving COJ at 1 year after KPE	*P*	Odds ratio (95% CI)	*P*
**Gender**					
Male	97	65 (67.0%)	0.574	0.81(0.43–1.38)	0.483
Female	134	89 (66.4%)		1	
**Associated major anomalies**					
Yes	17	10 (58.8%)	0.819	1.14 (0.74–1.86)	0.572
No	214	144 (67.2%)		1	
**Age at KPE (days)**					
< 51	60	41 (68.3%)	0.046	1.43 (0.92–1.93)	0.041
51 to 60	63	42 (66.7%)		1.67(1.45–1.85)	0.038
61 to 70	57	40 (70.2%)		1.89(1.76–1.99)	0.032
> 70	51	31 (60.8%)		1	
**COJ at 1 month after KPE**					
Yes	28	20 (71.4%)	0.228	1.58 (0.66–1.93)	0.071
No	203	134 (66.0%)		1	
**Adjuvant steroid therapy**					
Yes	169	115 (68.0%)	0.132	1.23 (0.72–1.64)	0.081
No	62	39 (62.9%)		1	

#### Native liver survival

**Figure 3:**
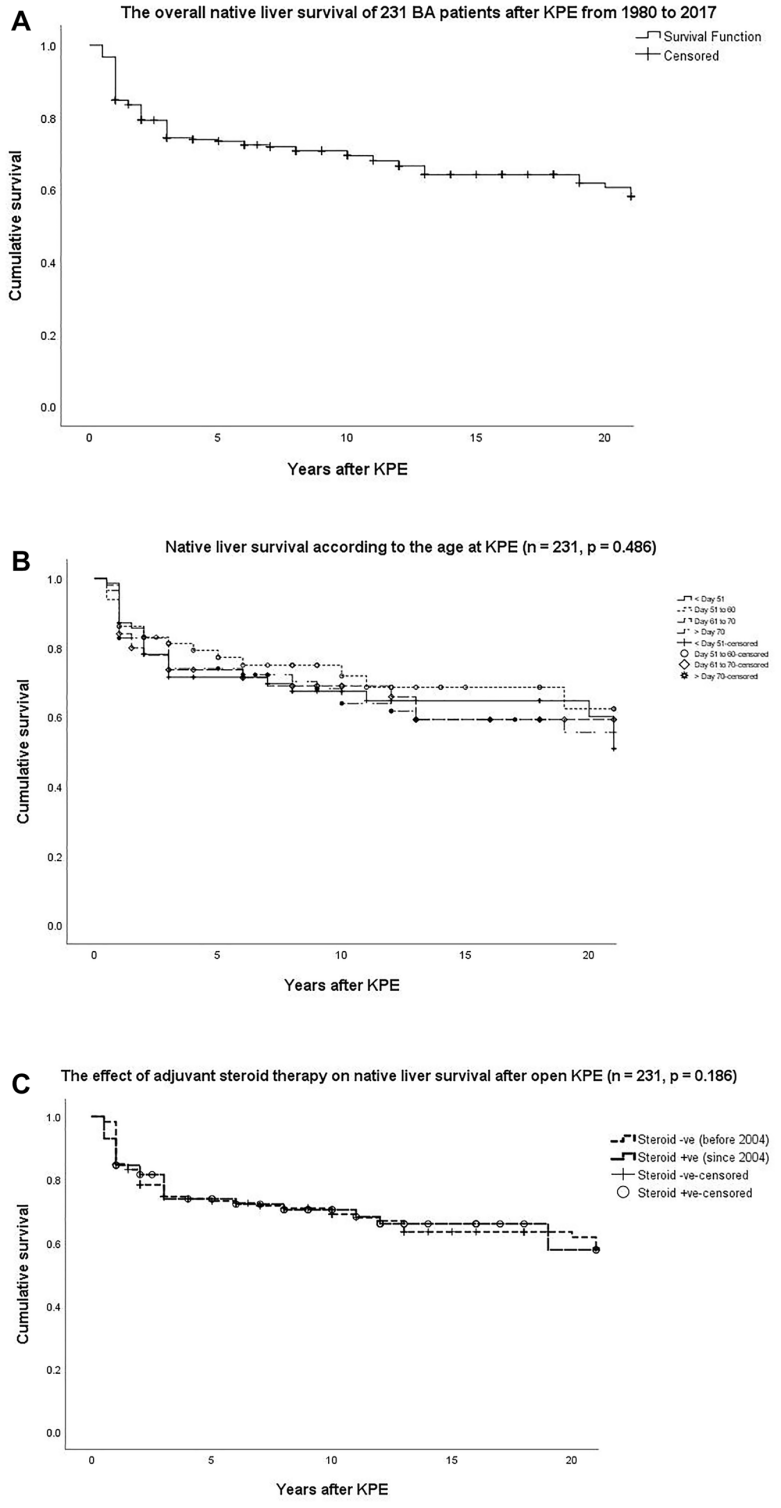
(**A**) The overall native liver survival of 231 BA patients after KPE from 180 to 2017. (**B**) Native liver survival according to the age at KPE (n = 231, *p* = 0.486). (**C**) The effect of adjuvant steroid therapy on native liver survival after open KPE (n = 231, *p* = 0.186).

Until the last follow up, 153 patients (66.2%) were still surviving with their own liver (Table 2). Forty-three of them (28.1%) have reached adulthood (≥ 18 years). Seventy-patients (30.3%) received LT at a median age of 6.2 (IQR: 4.3—8.4) years and 8 patients (3.4%) were recorded death after KPE without transplant. The indications for LT included: liver failure and the median Paediatric End-Staged Liver Disease (PELD)/Model for End-Staged Liver Disease(MELD) score was 16.4 (IQR: 13.5 – 24.3) (n = 59); severe portal hypertension (n = 7) and recurrent cholangitis (n = 4). Four patients presented with shortness of breath and were diagnosed with hepatopulmonary syndrome before LT. Kaplan–Meier analysis estimated the 10- and 20- year NLS rate were approximately 70.7% and 61.5%, respectively (Fig. 3A). There was no significant difference in the NLS rate when we compared the age at KPE and the use of adjuvant steroid therapy before and after 2004 (Fig. 3B,C).

### Clinical conditions of the native liver survivors (n = 153) (Table 3)

**Table 3:** The latest clinical conditions of the 153 native liver survivors of BA.

Clinical condition/blood parameter	Number (%) or median value (IQR)			
	Total (n = 153)	Jaundice + ve (n = 54)	Jaundice -ve (n = 99)	*P*
Growth				
Body weight centile (< 18 years)	56 (48–70)	54 (46–63)	57 (49–68)	0.451
Body height centile (< 18 years)	62 (53–72)	63 (53–69)	60 (53–75)	0.678
BMI (≥ 18 years)	24.2 (22.4–26.3)	23.6 (22.8–25.8)	25.1 (23.6–26.5)	0.382
Bilirubin level (µmol/L)	24 (16–36)	29 (24–35)	14 (10–16)	0.021
Albumin (g/L)	37 (32–42)	35 (31–39)	36 (34–40)	0.245
INR	1.1 (1.0–1.3)	1.0 (0.9–1.1)	1.1 (1.0–1.2)	0.488
Platelet* (× 10^9^)	145 (81–256)	135 (90–210)	152 (122–184)	0.503
Hypersplenism*	79 (51.6%)	35 (64.8%)	44 (44.4%)	0.369
OGV^	31/53 (58.4%)	–	–	–
Recurrent cholangitis*	42 (27.5%)	18 (33.3%)	24 (24.2%)	0.213

Jaundice is defined as total serum bilirubin level > 20 µmol/L.

*Percentage calculated based on 153 native liver survivors.

^As the percentage was calculated based on the 53 patients who have received upper endoscopy, the comparison between jaundice + ve and − ve was not performed.

#### Liver function and growth

Among 153 native liver survivors, 99 patients (64.7%) were jaundice-freed at the most recent follow up and the median bilirubin level of them was 24 (IQR: 16–36) µmol/L. Concerning the synthetic liver function, 94.8% and 91.5% of patients had a normal INR and albumin level for age. The median centile of body weight and height for patient younger than 18-year old were 56 (IQR: 48–70) and 62 (IQR: 53–72) respectively. For adult patients, the median body mass index was 24.2 (IQR: 22.4–26.3). When we compared the clinical conditions between patients with and without jaundice, there were no significant differences in their growth and liver synthetic functions.

#### Hypersplenism

All native liver survivors underwent ultrasound scan during follow up visit. Splenomegaly was found in 79 patients (51.6%). The median platelet count was 145 × 10^9^ (IQR: 81–256 × 10^9^) /L.

#### Oesophago-gastric varices (OGV)

At least one upper endoscopy was performed in 53 native liver survivors. The median age for the first endoscopy to be performed was 7 (IQR: 4–11) years. Among them, 31 patients (58.4%) were found to have OGV and the youngest patient was a 3 year old boy. Twelve patients experienced at least one bleeding episode requiring emergency endoscopic intervention.

#### Recurrent cholangitis

Forty-two native liver survivors (27.5%) suffered from more than one episode of cholangitis until the last follow up. The episode was regarded as severe in 13 patients who required antibiotics for more than two weeks. Their median bilirubin level upon admission was 52 (IQR: 26–212) µmol/L. Three patients with dilated intra-hepatic ducts required percutaneous transhepatic biliary drainage. After the removal of the drainage catheter, the three of them continued to survive without LT. Liver abscess were identified in 5 patients. While 3 patients responded to antibiotics therapy, 2 of them required image-guided drainage of pus. All of them recovered and remained transplant-freed. The median age to experience the first episode of cholangitis was 2 (IQR: 1.3–5.5) years. Five patients had their first cholangitic episode after adulthood.

## Discussion

The incidence of BA in our locality is close to other Asian countries and is slightly higher than the Caucasian population. However, we have a lower incidence of syndromic association^8–11^. We observed a higher incidence between 2006 and 2015 and this could be due to the transient immigration policy that allowed eligible women from Mainland China, one of the countries with the highest incidence of BA, to deliver their babies in Hong Kong during that period. Interestingly, although our incidence is higher than Western countries, we are not regarded as high-volume centres due to the small actual case number annually. To overcome the problem of limited patient volume, all BA patients in our public health care system are treated in the three centres in this study and the KPE always involved the experienced surgeons. Hence, the results of this cohort were relatively consistent across different eras when we compared the NLS before and after the introduction of adjuvant steroid therapy in 2004. This was achieved by the adherence to the same overarching surgical principle, the same peri-operative management and regular sharing of clinical experience among the three centres. The concentration of expertise in a small number of centres has enhanced our surgical outcomes with a 66.7% COJ rate achieved at the first year after KPE. Our analysis revealed that KPE before day 70 of life was associated with a higher chance of successful drainage. In this study, we did not identify any benefit associated with adjuvant steroid therapy. However, our steroid protocol was not standardized and hence we could not make a definitive conclusion. Herein, we included open KPE only because the results of laparoscopic KPE performed for a short period in one local centre were shown to be inferior to open KPE, a finding which corroborated with other international publications^12–14^. Nevertheless, some recent publications have re-evaluated the value of laparoscopic KPE and reported favourable outcomes^15,16^.

In recent years, BA has been transformed from a condition that used to be incompatible with life to a chronic disease with long-term complications^17^. Patients and their care-takers should be given adequate information about the possible post-operative complications and any non-specific but alarming symptoms, in order to facilitate early medical attention when complication arises. Among these, PHT is of upmost importance because there is still no effective therapy. There are surgical procedures reported for PHT in children^18,19^. However, except LT, most of them are not applicable for post-KPE patients. Nevertheless, in our series, only a minority of patients were transplanted for PHT. More than half of our survivors had hypersplenism but this may not always be symptomatic. In some patients, pancytopenia can develop despite their liver function being preserved and for this reason, complete blood count should be checked regularly. While hypersplenism maybe silent, OGV are prone to bleed. As there is no international consensus regarding surveillant endoscopy after KPE, there are variations in the policy among our centres. Only one third of our BA survivors (n = 53) have received endoscopic examination but there were already 31 (58.4%) cases of OGV detected; with 12 patients required emergency procedure for haemostasis. Given this high detection rate, we expect the actual number would be higher if all patients were surveyed. This finding also concurs with other studies reporting a high incidence of OGV detected by surveillance endoscopy^20,21^. Hence, in contrary to the belief of ‘on-demand’ endoscopy, we recommend that upper endoscopy should be performed in every BA patient to screen for OGV, preferably starting at 4 years after KPE. Prophylactic treatment by sclerotherapy or banding can be performed when OGV is detected. This policy should continue when the patient is transitioned to adult as OGV can continue to develop after adolescence. Even though PHT is considered as a predictor for future LT in a recent study by Jain et al.^22^, proper management of its complications could potentially keep them from LT. Recently, the value of liver stiffness measurement to monitor PHT has been reported and if available, this should be incorporated in the follow up protocol of BA patients^23^. Medical treatment for PHT are available and can be prescribed but the potency and side effect remains unknown due to the lack of large-scale clinical trials^24^.

Cholangitis is another potentially lethal complication after KPE and is postulated to be responsible for the worsening of cholestasis. In a study by Jain et al., a fourfold increase in the risk of future LT is found in patients with late onset of cholangitis^22^. Our data revealed that 27.5% of our patients had developed recurrent cholangitis, a figure lower than a recent study by a Korean group that reported 76.2%^25^. Notably, only a minority of patients in our series (n = 4) necessitated liver transplant solely due to the indication of recurrent cholangitis. The presentation could be subtle and sometimes it is difficult to differentiate it from flu. BA-related cholangitis is different from gallstone induced cholangitis in adult and abdominal pain may not be the chief complain. It could happen without an identifiable anatomical obstruction. All patients with clinical feature of cholangitis, namely fever and persistent jaundice, will undergo full work-up, followed by broad-spectrum antibiotics for at least 2 weeks to ensure adequate coverage. Clinically, in order to differentiate from common cold which may also lead to a mildly elevated bilirubin level, we labelled the episode as cholangitis only if jaundice persisted upon two blood taking. We believe an aggressive management approach to cholangitis is necessary to prevent it from becoming intractable that ultimately worsens the prognosis^26^. Liver abscess and ductal dilatation, though uncommon, should be actively sought out and external drainage can be performed if necessary. Even though most cholangitis occurred shortly after KPE, it can happen at any time and the first presentation can be at adulthood. Follow-up providers should therefore maintain a high index of suspicion in any BA survivors with fever and deranged liver function. Knowledge in this could expedite the treatment process without unnecessary investigations. Occasionally, long term prophylactic antibiotic is necessary to prevent recurrence. Home intravenous antibiotics administration has been reported with success especially in compact city where home-hospital distance is short^27^.

The value of KPE as the primary treatment has been challenged by the high post-Kasai LT rate (24). While it is still true that BA is the most common indication for paediatric LT, our survival analysis revealed that more than 60% of our patients could remain transplant-free as far as 20 years after KPE. This survival rate is comparable to the results reported by some high-volume centres^28,29^. We believe KPE should still be regarded as the primary treatment in experienced centres and LT should be regarded as a salvage treatment unless the presentation is late when KPE is bound to result failure. Nevertheless, the definition of late remains arbitrary and mostly it is a clinical judgement. From our data, the median bilirubin level among the native liver survivors was 24 µmol/L only and their growth was normal. There was no significant difference between those with and without jaundice. These findings, together with the conclusion from the previous study by Wong et al. on the quality of life among BA survivors^30^, has indicated that most post-KPE patients with mild cholestasis may not require LT if their complications are well managed and many can indeed survive with the disease. Regarding the optimal timing of LT after KPE, in our previous study focusing on all transplant recipients, we concluded that LT should be considered only when PELD/MELD score was greater than 15, taking into account the risk–benefit ratio^31^. The involvement of the paediatric surgical team in the long term management of these patients also facilitates the practice of personalized medicine due to a better knowledge in the patient’s history. For in-patient care, thanks to the geographical advantage in our city, patients could be easily transferred back to the parent hospital within a couple of hours for further management of disease-related complications. Under this management strategy, in addition to maximizing NLS, the timing of LT could also be prolonged to beyond 4 years, the median age for LT among BA patients previously reported by a multicentre study in the US^32^. We understand the threshold for listing a patient for LT could be different among different centres. Herewith, our purpose was to share our experience in how to avoid LT as long as possible when there is a scarcity of organ donors. Table 4 summarized our recommended management strategies for patients living with native livers after KPE.

**Table 4:** Recommended management strategies of patients living with native livers after KPE.

Liver failure
Patients should receive life-long follow up, preferably by the primary surgical teams who are knowledge in BA-related complications
Liver function and growth status should be checked every 3 to 6 months
Monitor PELD/MELD score and consider referral to liver transplant if the score is over 15
**Portal hypertension**
The first surveillance endoscopy should be performed starting from the fourth year after KPE to look for OGV
Treat OGV prophylactically by endoscopic injection sclerotherapy or banding
Instead of an ‘on-demand’ basis, endoscopy should be performed regularly
In addition to physical examination, ultrasonography should be performed regularly to monitor splenomegaly
If available, non-invasive measurement of liver stiffness should be carried out during the follow up
Medical treatment such as beta-blocker can be prescribed in symptomatic PHT but the potency and potential side-effects should be discussed
**Cholangitis**
Always maintain a high index of suspicion for possible cholangitis for any post-KPE patients admitted with fever and jaundice
Close monitoring for any clinical deterioration
An episode should be treated by potent antibiotics with adequate duration
In refractory cases, radiological imaging can be arranged to look for liver abscess or dilated intra-hepatic ducts that are treatable by interventional procedures
Consider long-term prophylactic antibiotics in patients with recurrent cholangitis

A major strength of our study is the relatively large sample size for a rare congenital disease as well as the availability of long-term data. However, we acknowledge there are several limitations to our study. First, 30 patients (12.9%) were excluded and their data could not be used. Second, the inconsistent policy regarding surveillance endoscopy and adjuvant steroid therapy have affected the analysis of their true clinical implications. Third, the broad spectrum of severity in cholangitis has precluded an in-depth analysis of the associated risk factors and its the impact on NLS. Lastly, due to the inherent retrospective nature of the study, some clinical information such as family history and microbiology results were not available.

In summary, the native liver survival of BA patients treated in a low- to mid- volume centre can be prolonged by the centralization of surgical care and a comprehensive follow up programme. The participation of the paediatric surgical team in the long-term management of BA patients enhances the delivery of precision care. While BA-related complications are common and clinically important, it is possible to avoid or defer LT with meticulous attention to them. Specifically, native liver survivors should be monitored closely for the development of PHT and cholangitis. OGV are common and early surveillance is therefore recommended to avoid life threatening bleeding. Post-Kasai patients with mild cholestasis and normal growth may not require LT. In places with shortage of organ donor, avoiding LT brings a remarkable benefit to the patients and the society. At patient level, children could be spared from a high risk ultra-major operation at young age and the use of immunosuppressants is delayed. To the society, the liver grafts which are valuable social resources, can be allocated to other liver failure patients and more lives will be saved.

## Abbreviations

BA: Biliary atresia
COJ: Clearance of jaundice
KPE: Kasai portoenterostomy
LT: Liver transplant
MELD: Model for end-stage liver disease
NLS: Native liver survival
OGV: Oesophago-gastric varices
PELD: Pediatric end-stage liver disease
PHT: Portal hypertension
